# Supplementary material for: Late Development of Cue Integration Is Linked to Sensory Fusion in Cortex
Source: Curr Biol. 2015 Nov 2;25(21):2856–61. doi: 10.1016/j.cub.2015.09.043 (PMC4635311; doi:10.1016/j.cub.2015.09.043)
Supplement: Document S1. Supplemental Experimental Procedures, Figures S1–S3, and Tables S1 and S2 [file mmc1.pdf]

Current Biology

Supplemental Information

Late Development of Cue Integration

Is Linked to Sensory Fusion in Cortex

Tessa M. Dekker, Hiroshi Ban, Bauke van der Velde, Martin I. Sereno, Andrew E. Welchman, and Marko Nardini

Figure S1, relates to Figure 3

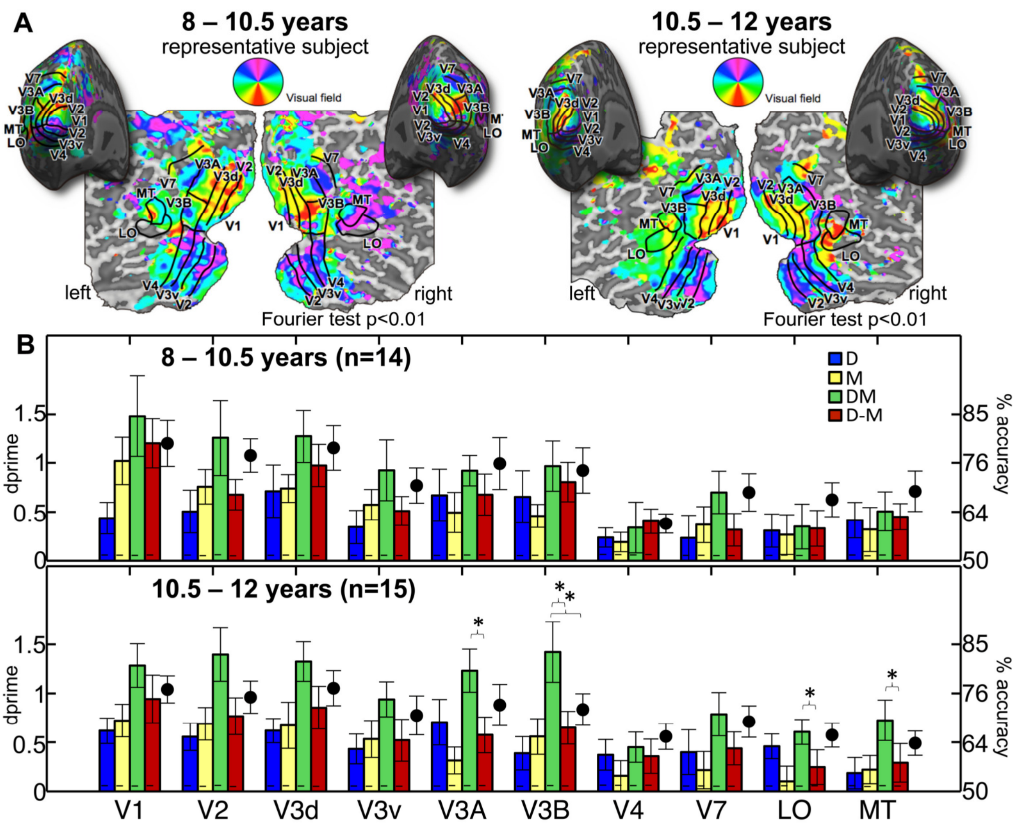

(**A**) Polar angle maps and borders of retinotopic regions of a representative younger and older subject are displayed on the same subjects' inflated and flattened cortical surfaces. (**B**) Depth decoding performance (d-prime and accuracy) for all retinotopic regions of interest is plotted per condition and age group. Error bars: 95%CI. Stars indicate p>0.05, Bonferroni-corrected, for description of analysis and p-values see Table S1.

We note that there is a (non-significant) trend for decoding for combined cues (DM) to be higher than for single cues (D or M) and conflicting cues (D-M) in visual ROIs along the cortical hierarchy – although only area V3B showed robust evidence for age-related changes in sensory fusion. This pattern differs somewhat from that seen by Ban et al. This may be (i) due to differences across experimental set-ups, or (ii) because aspects of sensory fusion are still developing between the ages of 10.5-12 years (as seen here) and adulthood (as reported previously). For example, initially, low-level areas may be recruited when children first start combining cues, and processing may gradually become more focused around area V3B. While we observe a clear shift in cortical function around the age when perceptual changes in sensory integration occur, it would be interesting to track development further, from age 10-12 years to adulthood to distinguish these possibilities.

Figure S2, relates to Figure 3

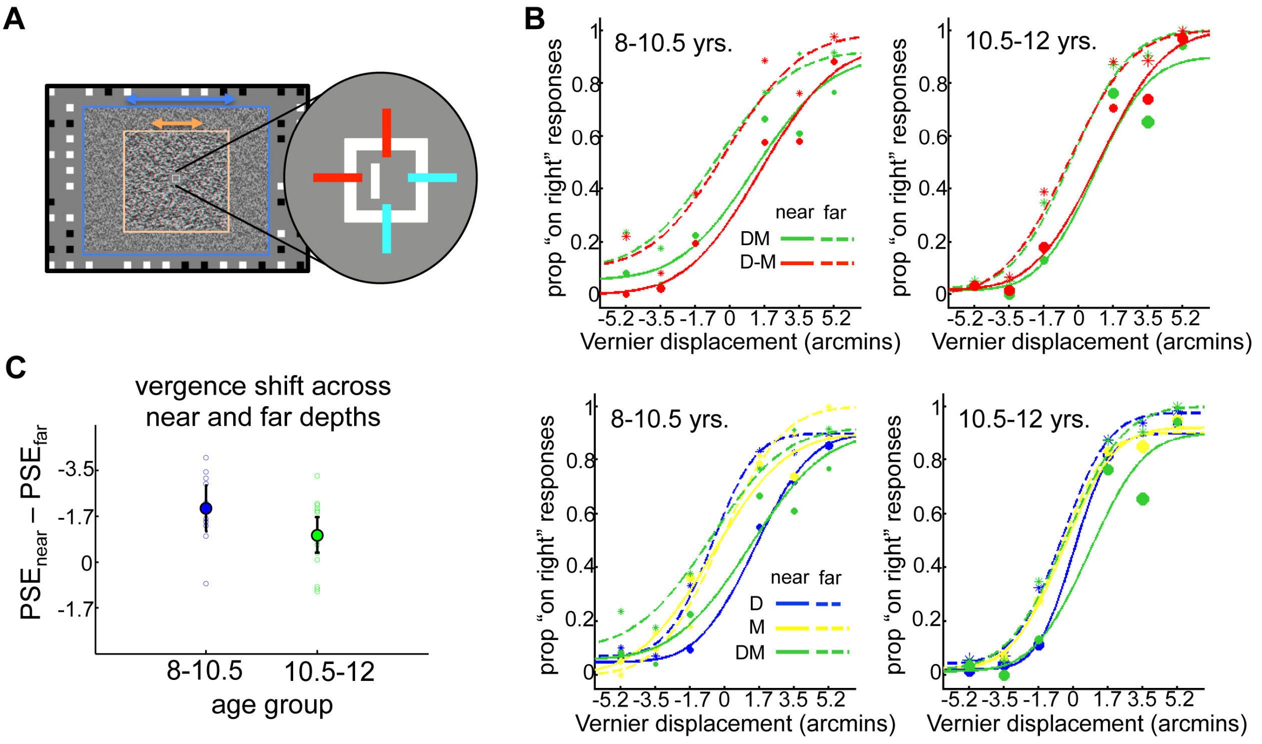

(**A**) During the Vernier task in the scanner, subjects judged whether targets (white stripe) appeared to the left or right of the top red nonius line. Targets appeared once every 4 trials (twice per block) on average, at three distances left or right of the centre. Because the red line is only presented to the left eye, a biased point of subjective equality (PSE, 50% “on the right” responses) indicates vergence in front of (positive) or behind (negative) the screen. (**B**) To compare vergence shifts across age, we fitted individual psychometric functions (Cumulative Gaussians) through responses for near and far planes collapsed across all conditions, and computed the difference in PSE for each individual (plotted, mean ± 95%CI). Four participants in the youngest group were excluded from the analysis, due to recording error (1) or because the functions fitted through their responses had a poor fit (GoF < 0.7). Subjects shifted their vergence across near and far stimuli by ~2 arcmins, a small proportion (~15%) of the total depth difference between the stimuli (12 arcmins). A trend for less vergence adjustment in the older age group did not reach statistical significance ($t(23)=1.83$, $p=0.079$). Crucially, there were no significant correlations between shifts in vergence and pattern classifier performance in any of the ROIs (largest Pearson’s $r=0.30$, $p=0.15$, in area V2, condition D), suggesting that vergence shifts did not drive voxel activation patterns, and hence are an unlikely explanation of our results. **(C)**

Because there were not enough datapoints, we could not fit functions for each condition separately for each subject. To ensure that collapsing near and far trials across conditions was valid (vergence shifts were not opposite for some conditions) we obtained psychometric functions per condition by collapsing across all subjects within each group. In both groups, the PSE shifted in a consistent way across near and far planes for the D, DM and D-M conditions (**B**, top and bottom plots). Vergence did not shift across depths defined by motion (yellow lines).

Figure S3, relates to Figure 3

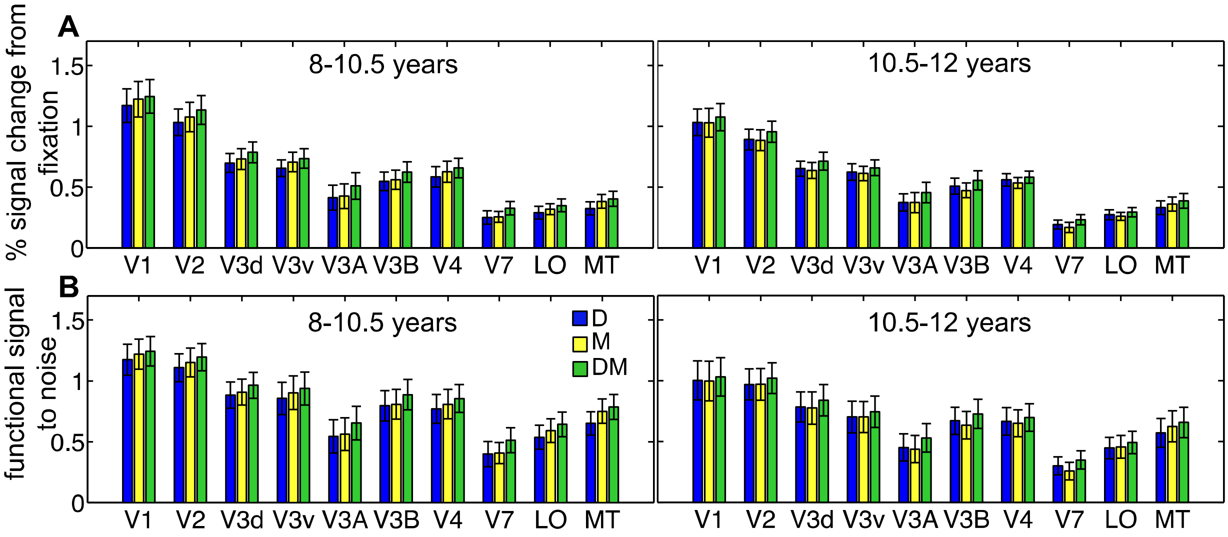

(**A**) Percent (%) fMRI signal change compared to the fixation baseline in the D, M and DM conditions, is visualized for each region of interest and age group (error bars: 95% CI). This was calculated from the mean response of the 400 voxels used for classification in each area. (**B**) To obtain a functional index of signal to noise, signal change was expressed in relation to the overall standard deviation of the signal $X_{stimulus}$- $X_{fixation}$/ $SD_{stimulus + fixation}$ (error bars: 95% CI). Both signal indices show a similar pattern: ROI (10) x Condition (3) x Age (2) ANOVAs revealed that both measures differed across stimulus conditions D, M and DM, (% signal change: $F(2,26)=29.5$, $p<0.01$; signal to noise: $F(2,26)=30.7$, $p<0.01$), with slightly higher signal for DM stimuli. Crucially, there were no significant main effects of Age (% signal change: $F(1,27)=2.1$, $p=0.16$; signal to noise: $F(1,27)=1.7$, $p=0.21$), or interactions of Age with Condition (% signal change: $F(2,26)=2.1$, $p=0.14$; signal to noise: $F(2,26)=1.9$, $p=0.17$); or with Age, Condition and ROI (% signal change: $F(5.9,156)=0.78$, $p=0.58$; signal to noise: $F(5.9,161.5)=0.39$, $p=0.88$). Furthermore, neither % signal change nor signal to noise were correlated with classifier performance in V3B, the area in which we found robust age differences in indices of sensory integration (largest Pearson's $r=0.13$, $p=0.50$). In sum, it is unlikely that age differences in our pattern classifier analysis were driven by differences in overall signal amplitude or functional signal to noise.

Table S1, relates to Figure 2.

Function Name	Description	Mean BIC
**Linear**	f(x) = p1*x + p2	-90.193
**Exponential**	**f(x) = p1*exp(p2*x)**	**-92.731**
**Quadratic**	f(x) = p1*x^2 + p2*x + p3	-92.179
**Two lines with inflection** Least square splines, 3 knots	Slope1=0, slope 2=free	-92.090
**Two lines with inflection** Least square splines, 3 knots	Slope1=free, slope 2=free	-88.980

To assess which function best captures the change in the integration indices in Figure 2A, we compared 5 plausible models: (1) a straight line, (2) an exponential increase, and (3) a quadratic change. We also fit the data with two straight lines connected by an inflection point using least square splines [S1], with (4) the slope of the first line set to zero (intercept, inflection-point and second slope free parameters), or (5) a freely varying first slope. For model comparison we used the Bayes Information Criterion, where the lowest value indicates the best-fitting model after model complexity is accounted for (i.e., models with more parameters are less parsimonious, so penalised). The best-fitting model was an exponential function. For DM vs. D-M: p1=$7.25*10^{-05}$ (95% CI: $-0.3152*10^{-4}$, $4.603*10^{-4}$) p2=0.6201 (95% CI: 0.1776, 1.062), R^2=0.10. For DM vs. $\sqrt{}D^2+M^2$: p1=$2.152*10^{-6}$ (95% CI: -2.43e-05, 2.861e-05), p2=0.8641, (95% CI: -0.1314, 1.86), R^2=0.15. The resulting curves (**black, Fig. 2A**) are in line with previous reports of no integration followed by integration in childhood [S2–S5], and with a gradual developmental process (not all children start integrating at exactly the same age). It is important to note, however, that model 3 (a quadratic function with a slight initial decrease followed by a steeper increase) and model 4 (a flat line connecting to an increasing slope) also had good fits to the data and led to similar conclusions that robust integration emerges around age 10.5 years.

Table S2, relates to Figure 3

	DM *vs.* D-M			DM *vs.* $\sqrt{M^2+D^2}$		
	p-values			*p-values*		
	<10.5 yrs	>10.5 yrs	age diff.	<10.5 yrs	>10.5 yrs	age diff.
V1	0.28	0.21	0.87	0.32	0.10	0.90
V2	0.057	0.022*	0.89	0.49	0.069	0.46
V3v	0.22	0.043*	0.59	0.58	0.094	0.56
V3d	0.12	0.0084*	0.99	0.46	0.18	0.97
V3A	0.086	0.0045*	0.09	0.66	0.035*	0.067
V3B	0.43	0.0022*	0.04*	0.80	0.0048*	0.036*
V4	0.76	0.49	0.52	0.85	0.37	0.78
V7	0.071	0.12	0.91	0.10	0.64	0.77
LO	0.94	0.0047*	0.20	0.10	0.77	0.11
MT	0.79	0.0040*	0.11	0.27	0.22	0.095

We tested which areas met the criteria for cue integration by comparing DM vs. D-M and DM vs. $\sqrt{(D^2+M^2)}$, using paired t-tests. We also tested if the two integration indices DM - D-M and DM - $\sqrt{(D^2+M^2)}$ differed significantly across age, using independent t-tests comparing index across age (results in table, * = $p<0.05$, underlined values survived Bonferroni correction). As explained in the main report, we specifically expected age-related changes in sensory fusion in area V3B (i.e. we had planned comparisons for this area based on three previously published studies). In younger children (<10.5 years), no area met both criteria for cue integration. In older children, area V3B met both criteria, and the age difference in both integration indices was significant (based on planned comparisons). We found a similar, but only marginally significant effect in area V3A. This is not surprising because V3A may have similar functional properties as V3B (since they are directly adjacent), and because slight inaccuracies in border identification may have miss-assigned parts of V3B to V3A.

Supplemental Experimental Procedures

Selection procedures for MRI

We first tested a large number of subjects between ages 6-12 years behaviourally in our lab and a local school (76; 50 of whom met inclusion criteria and are included in Fig 2). We observed an increase in integration between ages 8-12 years in this group. We then used identical procedures to test 67 more children in this smaller age range (53 of whom met inclusion criteria and are included in Fig 2), with the intention to select subjects for MRI. We invited 41 children back for fMRI (29 of whom met inclusion criteria - magenta data points in Fig 2, and data in Fig 3). They were selected based on several considerations: (1) parents *and* children were willing to take part after the experimenter explained what fMRI involved, (2) families were available during scanning slots within ~6 weeks of behavioural testing, (3) children met MRI safety criteria, (4) children appeared to not be very fidgety. We also attempted to (5) obtain a representative sample of each age group <10.5 & above >10.5 in terms of overall integration performance, and we aimed to (6) obtain age groups of equal size with good fMRI data quality (~15 subjects >10.5 years and <10.5 years).

To ensure that age differences were not driven by age-related confounds in attention, compliance, etc., we had to prioritise data quality over inclusion. The pattern of results stayed similar with more lenient exclusion criteria (see "Participants" section in methods). We judge it unlikely that a basic perceptual function (i.e., sensory fusion) is sensitive to any population sampling biases this may have caused.

Rewards for behavioral performance

To keep child participants motivated, focussed and entertained throughout the experimental tasks, they were shown their score at the end of each experimental block (or run inside the scanner), creating a game-like element. Scores were converted into coins that could be used to "buy" small prizes at the end of the session. The number of coins won was indicated by the number of smiley faces next to the interval in which the score fell along a score-bar from 0-100. Although participants were not informed of this, we computed scores based on the easiest trials only, to reward attention rather than individual differences in perceptual ability.

Procedures fMRI session 1

Before collecting pattern classification fMRI data, we collected a high-resolution structural scan and retinotopic data from each subject in a separate fMRI session. BrainVoyager QX (BrainInnovation B.V.) was used to transform each subject's T1-weighted 3D MPRAGE (1 mm^3 voxel size, Bandwidth=190 Hz/pix, 176 partitions, TR=8.4, TE=3.57, effective TI=1000 ms, flip angle=7 degrees) into Talairach space, inflate the cortex and create flattened surfaces of both hemispheres. We obtained retinotopic maps by measuring BOLD responses whilst subjects were presented with a rotating wedge stimulus overlaid with moving objects (polar angle mapper, cycles/run=6, eccentricity=~16°, 2 runs with clockwise, 2 counter-clockwise rotation). To stimulate fixation, subjects performed a central task: They detected brightness changes of the fixation dot during runs 1 and 2, and they detected letters briefly flashed on top of the dot during runs 3 and 4. We used a standard EPI sequence (TR=2.5s, volumes=132, slices=30 voxel size=3.2 cm^3, axial plane, interleaved, bandwidth=1930 Hz/pix, TE= 39 ms, flip=90).

Pre-procession steps session 1 and 2

Preprocessing steps for all functional data collected during MRI sessions 1 (retinotopic mapping) and 2 (depth cue integration), consisted of: initial volume removal (4 TRs), motion correction, slice time correction, linear trend removal and high-pass filtering (three cycles per run cut-off). We aligned functional runs to each subject's anatomical scan and both were transformed into Talairach space.

ROI selection

We used Fourier analyses and Fourier F-tests to obtain polar angle phase maps, which were then projected onto the subject's inflated cortical surface. Borders of visual areas of interest were identified for each individual following criteria derived from Ban et al. (2012). Previously, V3B/KO was mapped using a dedicated localiser. Due to limitations on scanning with young children, V3B was defined based on retinotopy alone. We identified the first appearance of upper field in superior visual cortex bordering V3, and estimated where the eccentricity minimum was between V3A and V3B [S6]. We then extended V3B down to the centre of gaze. This overlapped our V3B with an area that has been described as LO1 (e.g., [S7], and was activated by our V3B/KO localiser. The location of LO was informed by an object vs. scrambled object localiser Ban et al., 2012 did with adults, and follows [S8].

Details of Searchlight analyses

The searchlight analysis procedures are similar to those reported in [S9], to allow for comparison to adult data. Specifically, we performed the searchlight analysis in volume space for each individual by selecting spherical ROI with 8mm radii, moving voxel-wise through the entire scanned volume of cortex. To account for variability in spatial organisation across subjects, each accuracy map was then smoothed with a 3mm FWHM kernel to reduce inter-subject variability. We computed group-level t-value maps by testing where DM>D-M and DM>$\sqrt{(D^2+M^2)}$ (conjunctive $p<0.05$), and projected the result onto a representative cortical surface from each group for visualisation (shown in Figure 3). The results from the searchlight analysis are highly consistent with the results from the ROI analyses, which effectively factors out individual differences in retinotopic organisation. The results of older children (>10.5 years) are consistent with previous results from adults [S9]. When we aligned subjects into a common space based on gyral and sulcal patterns along the cortex [S10], respecting V3B ROI locations (following function-informed cortex-based alignment procedures described in Frost & Goebel, 2013), the searchlight results remained similar, with a potential slight shift towards V3A in the right hemisphere, consistent with marginally significant fusion effects in the V3A ROIs (see p. 5, and caption Figure 1S).

Supplemental References

1. D'Errico (2009). Matlab slmengine toolbox: http://www.mathworks.com/matlabcentral/fileexchange/24443-slm-shape-language-modeling.

2. Gori, M., Del Viva, M., Sandini, G., and Burr, D. C. (2008). Young children do not integrate visual and haptic form information. Curr. Biol. *18*, 694–698.

3. Nardini, M., Jones, P., Bedford, R., and Braddick, O. (2008). Development of cue integration in human navigation. Curr. Biol. *18*, 689–693.

4. Nardini, M., Bedford, R., and Mareschal, D. (2010). Fusion of visual cues is not mandatory in children. Proc. Natl. Acad. Sci. *107*, 17041.

5. Petrini, K., Remark, A., Smith, L., and Nardini, M. (2014). When vision is not an option: children's integration of auditory and haptic information is suboptimal. Dev. Sci. *17*, 376–387.

6. Press, W. A., Brewer, A. A., Dougherty, R. F., Wade, A. R., and Wandell, B. A. (2001). Visual areas and spatial summation in human visual cortex. Vision Res. *41*, 1321–1332.

7. Larsson, J., and Heeger, D. J. (2006). Two retinotopic visual areas in human lateral occipital cortex. J. Neurosci. Off. J. Soc. Neurosci. *26*, 13128–13142.

8. Malach, R., Reppas, J. B., Benson, R. R., Kwong, K. K., Jiang, H., Kennedy, W. A., Ledden, P. J., Brady, T. J., Rosen, B. R., and Tootell, R. B. (1995). Object-related activity revealed by functional magnetic resonance imaging in human occipital cortex. Proc. Natl. Acad. Sci. *92*, 8135–8139.

9. Ban, H., Preston, T. J., Meeson, A., and Welchman, A. E. (2012). The integration of motion and disparity cues to depth in dorsal visual cortex. Nat. Neurosci. *15*, 636–643.

10. Fischl, B., Sereno, M. I., Tootell, R. B., and Dale, A. M. (1999). High-resolution intersubject averaging and a coordinate system for the cortical surface. Hum. Brain Mapp. *8*, 272–284.
