# Supplementary material for: Late Development of Cue Integration Is Linked to Sensory Fusion in Cortex
Source: Curr Biol. 2015 Nov 2;25(21):2856–61. doi: 10.1016/j.cub.2015.09.043 (PMC4635311; doi:10.1016/j.cub.2015.09.043)
Supplement: Document S2. Article plus Supplemental Information [file mmc2.pdf]

Report

Current Biology

Late Development of Cue Integration Is Linked to Sensory Fusion in Cortex

Highlights

- Children aged 6–12 years viewed displays depicting depth via two visual cues
- We used MVPA fMRI to test for fusion of these cues in retinotopic cortex
- Perceptual and cortical fusion of depth cues emerged only after age ~11 years
- Children's perception is limited by immature fusion of depth cues in visual cortex

Authors

Tessa M. Dekker, Hiroshi Ban, Bauke van der Velde, Martin I. Sereno, Andrew E. Welchman, Marko Nardini

Correspondence

t.dekker@ucl.ac.uk

In Brief

Humans combine sensory signals to improve perceptual precision, but this ability does not develop until ~11 years of age. Dekker et al. show that the onset of this perceptual skill is linked to functional changes within the visual cortex, revealing the surprisingly long development of sensory mechanisms underlying human perception.

CrossMark

Dekker et al., 2015, Current Biology *25*, 2856–2861
November 2, 2015 ©2015 The Authors
http://dx.doi.org/10.1016/j.cub.2015.09.043

CellPress

CellPress

Current Biology
Report

Late Development of Cue Integration Is Linked to Sensory Fusion in Cortex

Tessa M. Dekker,[1,*] Hiroshi Ban,[2,3] Bauke van der Velde,[1] Martin I. Sereno,[4,5] Andrew E. Welchman,[6] and Marko Nardini[1,7]
[1]Institute of Ophthalmology, University College London, 11-43 Bath Street, EC1V 9EL London, UK
[2]Center for Information and Neural Networks, National Institute of Information and Communications Technology, 1-4 Yamadaoka, Suita, Osaka 565-0871, Japan
[3]Graduate School of Frontier Biosciences, Osaka University, 1-3 Yamadaoka, Suita, Osaka 565-0871, Japan
[4]Birkbeck, University of London, Malet Street, WC1E 7HX London, UK
[5]Psychology and Language Sciences, University College London, 26 Bedford Way, WC1H 0AP London, UK
[6]Department of Psychology, University of Cambridge, Downing Street, CB2 3EB Cambridge, UK
[7]Department of Psychology, Durham University, South Road, DH1 3LE Durham, UK
*Correspondence: t.dekker@ucl.ac.uk
http://dx.doi.org/10.1016/j.cub.2015.09.043
This is an open access article under the CC BY license (http://creativecommons.org/licenses/by/4.0/).

SUMMARY

Adults optimize perceptual judgements by integrating different types of sensory information [1, 2]. This engages specialized neural circuits that fuse signals from the same [3–5] or different [6] modalities. Whereas young children can use sensory cues independently, adult-like precision gains from cue combination only emerge around ages 10 to 11 years [7–9]. Why does it take so long to make best use of sensory information? Existing data cannot distinguish whether this (1) reflects surprisingly late changes in sensory processing (sensory integration mechanisms in the brain are still developing) or (2) depends on post-perceptual changes (integration in sensory cortex is adult-like, but higher-level decision processes do not access the information) [10]. We tested visual depth cue integration in the developing brain to distinguish these possibilities. We presented children aged 6–12 years with displays depicting depth from binocular disparity and relative motion and made measurements using psychophysics, retinotopic mapping, and pattern classification fMRI. Older children (>10.5 years) showed clear evidence for sensory fusion in V3B, a visual area thought to integrate depth cues in the adult brain [3–5]. By contrast, in younger children (<10.5 years), there was no evidence for sensory fusion in any visual area. This significant age difference was paired with a shift in perceptual performance around ages 10 to 11 years and could not be explained by motion artifacts, visual attention, or signal quality differences. Thus, whereas many basic visual processes mature early in childhood [11, 12], the brain circuits that fuse cues take a very long time to develop.

RESULTS

To measure how cue integration develops in childhood, we adapted methods used with adults [3]. We presented dot displays depicting a target square in front of or behind its surround (Figure 1A). The impression of depth was created using differences in dot positions between the two eyes (binocular disparity) and differences in the target's speed relative to its surround (relative motion). Using this disparity-motion stimulus space, we created four conditions in which the two targets' near versus far depth was defined by (1) disparity, D, where the motion cue indicated zero depth (i.e., flat); (2) relative motion, M, where the disparity cue was flat; (3) both cues conveying consistent depths, DM (e.g., disparity = "near"; motion = "near"); or (4) both cues in extreme conflict, D-M (e.g., disparity = "near"; motion = "far").

These stimuli were designed to distinguish between two possible detection mechanisms. An optimal *fusion/integration* mechanism averages disparity and motion depth estimates into a fused estimate with lower variance (Figure 1A, left; the fused distributions are more sharply peaked). Under this scenario, the stimuli are more discriminable because fused depth estimators are more reliable. Alternatively, we can conceive an optimal *independence* mechanism that exploits the outputs of separate detectors for disparity and motion. This mechanism works by finding the maximal separation between the two stimuli (i.e., the magenta and cyan "blobs" in Figure 1A are furthest apart when projected orthogonal to the negative diagonal). Performance for this mechanism corresponds to the quadratic sum of the separations along the disparity and motion dimensions, which makes intuitive geometrical sense in terms of Pythagoras' theorem. Under this scenario, stimuli are more discriminable when defined by two cues because their effective separation is increased ($DM = \sqrt{[D^2+M^2]}$). Typically, the performance of these mechanisms looks very similar; however, we can distinguish them experimentally in two ways.

First, both mechanisms will be more sensitive when depth is defined by two cues in agreement (DM condition) compared to depth defined by the single cues (D or M). However, the fusion mechanism is less sensitive when cues are in conflict (D-M),

because opposing depth values are averaged together. By contrast, as the independence mechanism uses detectors that only measure one aspect of the stimuli (i.e., only depth from motion or only depth from disparity), the depth sign is effectively ignored (Figure 1B, criterion 1), i.e., the Pythagorean separation still increases whether the cues agree or disagree.

Second, we can compare performance in the DM condition with an ideal observer prediction. For an independence mechanism, this is the quadratic sum of performance in the "single cue" conditions (D and M). However, the fusion mechanism is compromised in the single cue conditions: e.g., the "flat" disparity cue is averaged with the near or far motion cue, resulting in smaller differences between near versus far stimuli. In the fusion case, as the quadratic sum prediction uses (compromised) D and M performance, empirical performance in DM (where all conflict is removed) will surpass the prediction (Figure 1B, criterion 2; see [13] for more details).

Behavioral Psychophysics Measures of Cue Integration

To assess perceptual cue integration, children (n = 103; age 6–12 years) judged which of two sequentially presented square planes appeared "furthest" behind the surround (Figure 1A). We fit their responses with a cumulative Gaussian and quantified performance using 1/sigma (larger indicates better depth sensitivity). We measured performance under the four experimental conditions (D, M, DM, and D-M) and assessed integration using our two criteria (1) DM − D-M and (2) DM − $\sqrt{(M^2+D^2)}$ (Figure 2A; positive values suggest sensory fusion).

Both indices increased between the 6th and 12th year of life (Pearson's linear correlation DM − D-M = 0.25; DM − $\sqrt{[M^2+D^2]}$ = 0.28; both $p < 0.05$). This increase was well described by an exponential function, diverging from zero around age 10 years (Figure 2A; for details and model selection, see Table S1). As a group, however, 10 year olds did not meet the integration criteria yet whereas 11 year olds did (yellow circles and 95% CIs are above zero). We therefore identified 10.5 years as a reasonable (although necessarily approximate) cutoff age for the emergence of robust integration abilities. Indeed, children grouped into an age bin of 10.5–12 years (Figure 2B) met both criteria for perceptual cue integration (paired t tests of DM versus D-M: t_{41} = 3.8, $p < 0.001$; DM versus $\sqrt{[M^2+D^2]}$: t_{41} = 3.3, p = 0.002). By contrast, we found no evidence for integration at 6–8.5 years (DM versus D-M: t_{20} = 1.6, p = 0.13; DM versus $\sqrt{[M^2+D^2]}$: t_{20} = −0.71, p = 0.49) or 8.5–10.5 years (DM versus D-M: t_{37} = 0.15, p = 0.88); DM sensitivity is *lower* than $\sqrt{(M^2+D^2)}$ prediction: (t_{39} = −2.3; p = 0.034). This is not because relative motion was too unreliable compared with disparity to induce perceptual benefits via fusion; M and D differed most in 10.5–12 year olds, where the motion cue nevertheless induced fusion. Together, these psychophysical data show that adult-like perceptual benefits from integrating disparity and motion cues to depth only become reliable by 10 or 11 years of age. Note, whereas our tests uncover integration, we cannot test

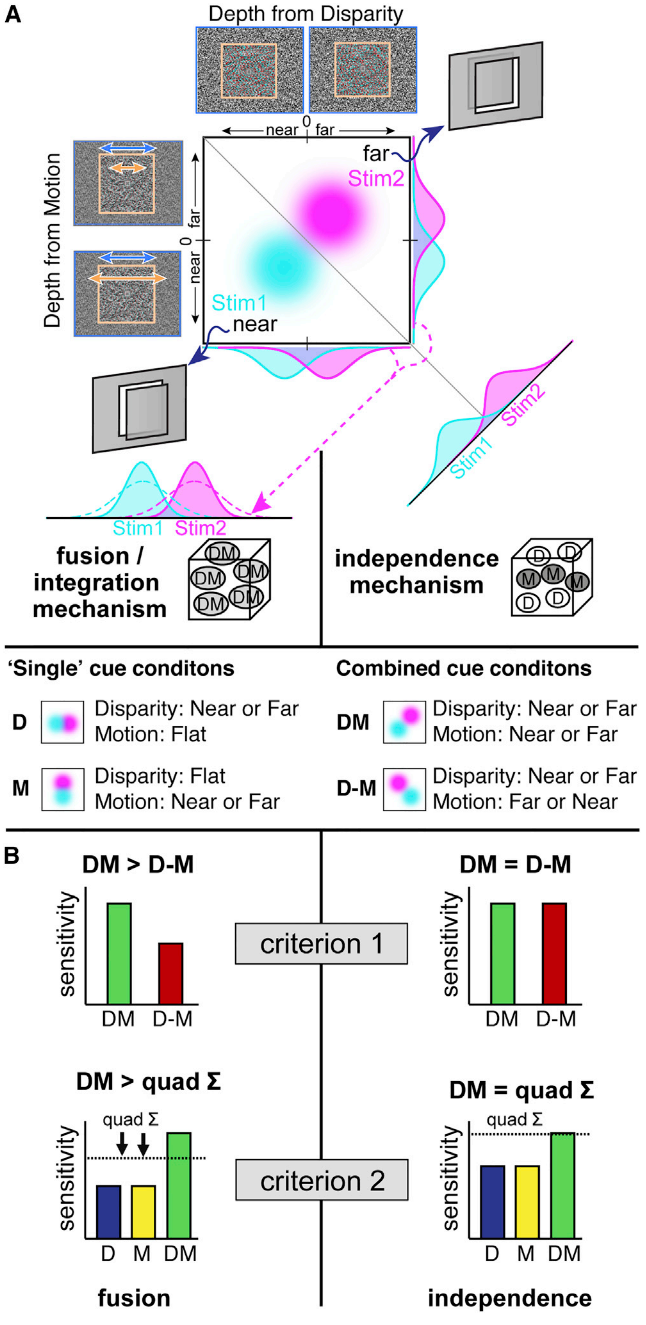

Figure 1. Detection Mechanisms and Integration Criteria

(A) Near versus far depths defined by motion and disparity. We illustrate depth estimators for stimulus 1 versus 2 as bivariate Gaussian distributions (magenta versus cyan "blobs") in this motion-disparity space. A fusion mechanism (left) combines the cue dimensions to reduce variance: averaged estimator distributions become more "peaked" and are thus more discriminable. By contrast, an independence mechanism (right) increases the effective separation between the stimuli: by Pythagoras' theorem (quadratic sum), the peak-to-peak separation between stimulus 1 and 2 is greater along the hypotenuse. Experimentally, we draw stimuli from different regions of the disparity-motion space. In "single" cue cases (D and M), stim1 and 2 differ in disparity-defined depth, but motion stays the same or vice versa. In combined cue conditions, disparity and motion can depict depth positions consistently (DM) or indicate opposite depths (D-M).
(B) Predictions for fusion (left) versus independence (right) mechanisms. In *criterion 1*, the fusion mechanism is compromised (lower performance) in the D-M condition, but the independence mechanism is unaffected because depth differences are detected independently. In *criterion 2*, the fusion mechanism is compromised by the "flat" cues in the D and M single cue conditions. In consequence, the ideal observer prediction (quadratic sum) underestimates DM empirical performance in the fusion case.

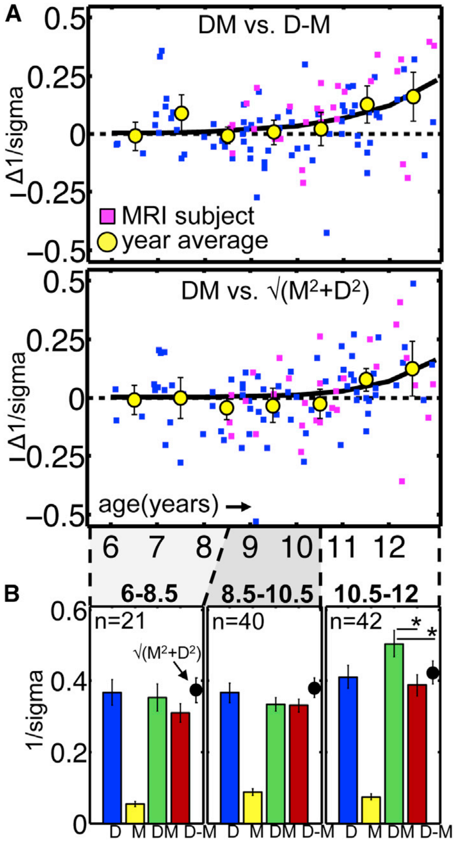

Figure 2. Behavioral Psychophysics Results

(A) Integration indices by age: individual subjects and mean by year (error bars: 95% CI), and exponential function fitted to individual data points. Positive values indicate cue integration.

(B) Mean (95% CI) 1/sigma of cumulative Gaussians fitted to participants' depth discriminations. Higher values indicate better depth sensitivity.

whether integration is optimal. Previous behavioral work with children tested for optimal fusion [9] by isolating cues from each other [1], but the stimulus differences required create interpretational difficulties for fMRI, so our single cue conditions were designed to contain cue conflicts.

fMRI Measures of Cue Integration

We measured fMRI BOLD responses in independently localized retinotopic regions of interest (scanned volume, Figure 3A; ROIs, Figures 3C and S1A; Supplemental Experimental Procedures) in the visual cortex of 8–12 year olds (n = 29; magenta symbols in Figure 2A). We presented near or far depth stimuli under the four experimental conditions while participants performed an orthogonal fixation task that required no depth judgements (see Control Analyses; Figure S2). We analyzed the data in each ROI by testing the performance of a linear support vector classifier trained to predict near versus far depth based on voxel activation patterns evoked when participants viewed targets in the D, M, DM, and D-M conditions. As with the psychophysical measures, we compared prediction accuracies using two criteria for cue fusion: DM > D-M (criterion 1) and DM > $\sqrt{(D^2+M^2)}$ (criterion 2).

Adults show evidence for integrated depth representations in cortical area V3B [3–5] (overlapping with the kinetic occipital [KO] area [13], a region of cortex also designated LO1) [14]. We therefore expected the development of sensory fusion to be expressed in this brain area. In younger children (<10.5 years), there was no evidence for integration in V3B (Figure 3; paired t tests of DM versus D-M: t_{13} = 0.82, p = 0.43; DM versus $\sqrt{[D^2+M^2]}$: t_{13} = 0.26, p = 0.80). By contrast, in older children (>10.5 years), V3B activation patterns met our criteria for integration: depth decoding for congruent depth stimuli exceeded performance for conflicting depth stimuli *and* single-cue predictions (Figure 3; DM versus D-M: t_{14} = 3.7, p = 0.002; DM versus $\sqrt{[D^2+M^2]}$: t_{14} = 3.3, p = 0.005). These differences in V3B response across age groups were statistically significant (independent samples t tests: DM − D-M, t_{27} = 2.1, p = 0.043; DM − $\sqrt{[D^2+M^2]}$, t_{27} = 2.2, p = 0.036). The age-related change in integration occurred despite decoding accuracies for the D and M conditions being similar across groups (D: t_{27} = 1.18, p = 0.25; M: t_{27} = −0.64, p = 0.527) and well above chance (black dotted lines, Figure 3).

We also tested for sensory fusion in V1-4, V3A, V7, LO, and MT (Figure S1B; Table S2; V1 in Figure 3). However, none of these other areas met both criteria for cue integration in either younger or older children. Specifically, indices of integration did not differ across age in any region besides V3B (Figure S1B; Table S2; there were marginally significant effects in directly adjacent area V3A). To ensure we had not missed areas outsides our localized ROIs, we ran a group-level searchlight analysis looking for areas where DM > D-M and DM > $\sqrt{(D^2+M^2)}$. Results (Figure 3) are mapped onto a representative cortex from each age group for visualization. No cortex met the criteria for integration in 8–10.5 year olds. In older children, only cortex around area V3B met both criteria. Thus, whereas depth defined by motion and disparity could be decoded reliably across visual cortex (including V3B) at all ages, robust evidence for fusion of these cues only emerged around ages 10 to 11 years, coinciding with marked improvements in behavioral performance (Figure 3B, small graphs). This suggests an area intricately linked to the development of cue integration but does not imply a sole locus of fusion nor exclude the involvement of other areas outside the sampled volume.

Control Analyses

We took precautions to minimize age-related confounds. Control analyses suggest that our fMRI findings cannot be explained by motion artifacts, visual attention, or signal quality. First, after our stringent movement exclusion criteria (see Experimental Procedures) head movements were small and equivalent across age groups and did not correlate with classifier performance (*mean [and SD] scan-to-scan displacement* < 10.5 years: 0.038 [0.014] mm; >10.5 years: 0.029 [0.014] mm, t_{27} = 1.61, p = 0.12; *mean absolute scan-to-scan rotation* < 10.5 years: 0.011° [0.0041]; >10.5 years: 0.0085° [0.0040], t_{27} = 1.73, p = 0.095). Second, to control eye vergence and fixation during fMRI, subjects performed left/right Vernier discriminations with small infrequent targets flashed briefly with 25% probability. Shifts in perceived position provided a subjective index of vergence [15] and revealed a slight change in fixation depth across near and far stimuli (~2 arcmin; 15% of the stimulus depth). This shift

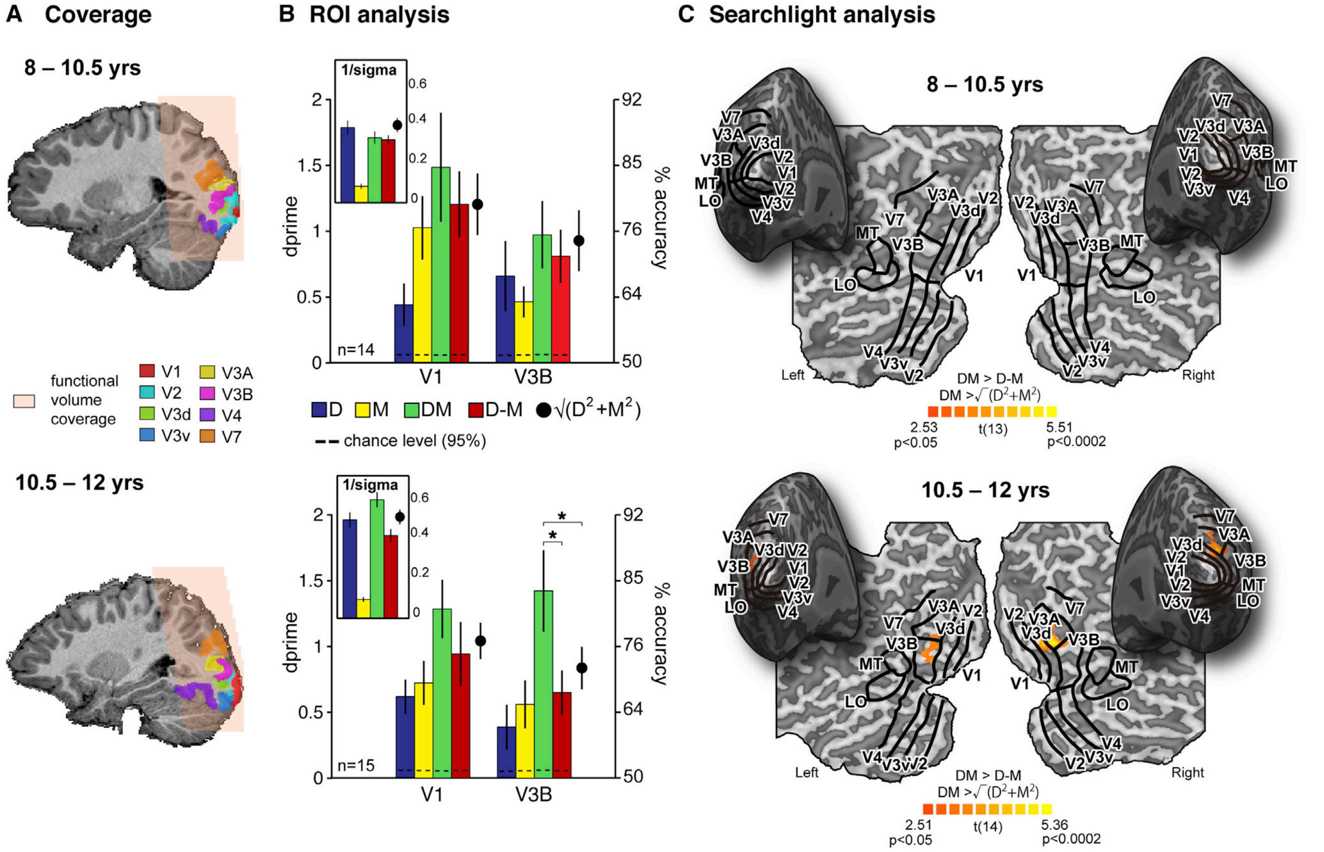

Figure 3. Pattern Classification fMRI Results

(A) Scanned area and ROIs for two representative subjects

(B) Large bar plots: accuracy (d-prime) with which near versus far stimulus depth was decoded from V3B activation patterns. V1 is shown for comparison. Performance was well above chance level; upper bounds (97.5%) were derived from permutation tests (dashed lines). Small panels show the same subjects' perceptual performance (1/sigma).

(C) Searchlight results. Individual accuracy maps were smoothed (3 mm FWHM) to account for inter-subject variability before statistical maps were computed and projected on the inflated cortical surface. ROIs from two representative subjects are super-imposed on the group result flatmaps. Note that significant regions may therefore be slightly misaligned with respect to their labels. Areas where both integration indices were significantly above zero are colored. T values are from DM versus $\sqrt{(D^2+M^2)} > 0$.

did not correlate with prediction accuracy (Figure S2), making fixation differences an unlikely explanation of our findings. Whereas the proportion of Vernier targets responded to (correctly *and* incorrectly) differed slightly across condition ($F_{7,17} = 2.4$; $p = 0.035$; due to more responses in D_{far} than D_{near}), this was similar across group ($F_{7,17} = 0.82$; $p = 0.58$), suggesting shifts in vigilance across condition did not vary with age. Finally, there were no age differences in percent signal change and functional signal to noise (signal mean/SD; Figure S3) or in overall SVM prediction accuracy (<10.5 years: 65% [SD = 6.1]; >10.5 years 65% [SD = 5.6]; $t_{27} = 0.57$; $p = 0.57$), which suggests that data quality and attention to display were well matched across groups.

DISCUSSION

Our findings reveal striking changes in depth representations within visual cortex until late childhood. This suggests that delays in sensory integration found at the behavioral level have their roots in the late maturation of neural circuits involved in sensory fusion, rather than downstream, post-perceptual decision processes.

Does this reflect the need for extensive perceptual learning about cues before they can be combined or a long postnatal time course for human brain maturation? Animal studies indicate an important role for learning: dark-reared kittens fail to develop neural markers of integration without exposure to audio-visual cue pairs [16]. When cue pairs were provided—but misaligned in space—emerging audio-visual integration responses in superior colliculus reflected this unusual relationship [17]. This suggests experience is crucial for learning when cues should be integrated. By extension, it is possible that children in our study did not integrate depth cues until 10 to 11 years because they were still learning to assign the cues to a single common cause. However, this is unlikely as disparity and relative motion both index relative distance across the retina (one across eyes; the other within eyes over time) and are typically highly correlated [18, 19]. Therefore, if the need to learn when these cues jointly signal

depth were the only obstacle to developing sensory fusion in childhood, we might expect integration to develop relatively early for this cue pair. Instead, the developmental time course we found resembles those of other cues, both within and across modalities [8, 9, 20], raising the possibility of a maturational bottleneck. Accordingly, once correlated audio-visual signals are provided to mature visually deprived cats, super-additive audio-visual integration responses in superior colliculus develop much faster than in young animals [21]. This suggests that the normal time course is not only prolonged by the need to learn cue statistics but also by constraints from neural circuitry. Whereas it is not yet clear how this relates to human development, the human brain undergoes substantial changes in long- and short-range myelination and connectivity in late childhood [22–24]. It is possible that such changes play a role in the development of cue integration abilities, for example, by segregating (decorrelating) sensory pathways, thus improving the efficiency of a fusion process [25].

More generally, our findings suggest that perception in childhood is not only limited by noisier signal processing [26, 27]. Rather, the developing brain is still optimizing how it represents and combines uncertain information to make inferences about the world [28]. Current models of brain function place this process at the core of perception and cognition [29, 30]. Other perceptual skills that are still developing in late childhood such as object [31–33] and scene perception [34] may also be affected by the suboptimal detection of patterns in noise. This highlights the need to understand the extent to which perceptual development in general may be described as optimization of inference.

EXPERIMENTAL PROCEDURES

Participants

We assessed depth discrimination in 142 children aged 6–12 years with no known visual or neurological problems; 27 withdrew from testing or had difficulty perceiving depth from disparity and 39 were tested but excluded from analysis because psychometric functions fitted to their data had a poor fit ($R^2 < 0.7$), showed a large bias (>3 arcmin), or integration indices (Figure 2A) deviated >5 SDs from the mean. We report psychophysical measures from 103 children (for age distribution, see Figure 2A). Less-stringent exclusion criteria ($R^2 < 0.65$; bias > 99; $N_{included} = 112$) did not change the results. Forty-one 8–12 year olds were invited back for retinotopic mapping and depth cue integration fMRI sessions. After exclusions due to excessive movement (n = 8), failure to complete both sessions (n = 2), or unclear retinotopic borders (n = 1), we report fMRI results of 29 children (magenta symbols, Figure 2A). See Supplemental Experimental Procedures for MRI selection procedures. Procedures were approved by the UCL Research Ethics Committee.

Stimuli and Task

Stimuli consisted of random dot stereograms rendered for red/cyan anaglyphs (following [3]), presented against a mid-gray background (Figure 1A). A central target square (11° × 11°) defined by relative motion and/or binocular disparity was surrounded by a "background" rectangle (20° × 16°) located in the plane of the projection screen. Both dot planes moved horizontally with a sinusoidal movement period of 1 s. The background movement amplitude was fixed at 0.5°. Condition orders were randomized. We accounted for interocular distance and calibrated displays to minimize crosstalk through the anaglyph glasses. Performance was rewarded (Supplemental Experimental Procedures).

Behavioral Psychophysics

Two depth stimuli were presented for 1 s each, separated by a 0.5-s interval fixation screen. Participants judged which of the two intervals contained the square furthest behind its background. One interval had a fixed depth (disparity: 8 arcmin and/or movement amplitude: 0.25°) whereas the other varied across six levels (3–13 arcmin disparity and/or 0.1°–0.4° of movement). In the extreme conflict condition (D-M), relative movement depicted the target in front of the plane (fixed target depth: 0.75°; variable target depths: 0.6°–0.9°). D, M, DM, and D-M stimuli were presented in blocks of 90 trials (15 trials per six depth levels). Participants practiced discriminating the largest depth difference correctly four times in a row before starting each block. Stimuli were presented on an LCD screen (1,920 × 1,080 pix) at eye height and 50 cm distance at 60 Hz.

fMRI

In the scanner, stimuli were either in front of (near; disparity −6 arcmin; motion 0.75°) or behind (far; disparity +6 arcmin; motion 0.25°) the surround. The Vernier fixation stimulus was a square (0.5° × 0.5°) with horizontal and vertical nonius lines (0.35°). The fixation marker was presented within a 1° cut out in the stimulus, at the same (zero) disparity of the projection screen. There were eight stimulus configurations: two target depths (near and far) for each experimental condition (D, M, DM, and D-M). These were presented in blocks of eight stimuli, each shown for 1 s with a 1-s fixation interval. Runs consisted of three repetitions per condition and a 16-s fixation baseline at the start and end. We collected six runs per participant. Stimuli were back projected (1,920 × 1,080 pix) onto a screen inside the bore of the magnet; viewing distance was 65 cm.

MRI Methods

Imaging

BOLD measures were obtained using single-shot EPI (TR = 2 s; volumes = 128; slices = 22; voxel size = 2.3 mm^3; isotropic) with a Siemens 1.5T MRI scanner and 32-channel coil without top. Participants who moved more than 1 mm or 5° from scan to scan *at any point* were excluded from the analysis. In a separate session, we collected high-resolution structural scans and identified retinotopic regions using polar angle mapping (Figure S1A). Standard preprocessing was done with BrainVoyagerQX (Supplemental Experimental Procedures).

Multi-voxel Pattern Analysis

For the ROI analysis, we selected the 400 most-responsive gray matter voxels in each region, based on the *t* statistic resulting from contrasting all stimulus conditions with the fixation baseline. For the searchlight classification analysis [35] (Supplemental Experimental Procedures), we selected spherical ROI with 8-mm radii, moving voxelwise through the volume of cortex. Voxel time courses were converted to *Z* scores and shifted by two TRs (4 s) to account for the hemodynamic response. To create voxel patterns, we averaged all eight volumes within each block. Each pattern was then mean centered by subtracting the mean voxel amplitude. We used a linear support vector machine (penalty parameter C = 1) [36] to classify near versus far depth stimuli in each condition (D, M, DM, and D-M) using a leave-one-out cross-validation procedure with six folds, with 15 near and far patterns in each training and three in each test. Mean prediction accuracies were converted to *d*-prime using:

$$d - \text{prime} = 2 * \text{erfinv}(2 * \text{accuracy} - 1);$$

erfinv, inverse error function. We ran permutation tests to assess chance level prediction accuracy for these data (dotted lines, Figures 3B and S1B) by running 1,000 SVMs with shuffled near/far labels.

SUPPLEMENTAL INFORMATION

Supplemental Information includes Supplemental Experimental Procedures, three figures, and two tables and can be found with this article online at http://dx.doi.org/10.1016/j.cub.2015.09.043.

ACKNOWLEDGMENTS

Supported by UK ESRC grant RES-061-25-0523, the Wellcome Trust (095183/Z/10/Z), JSPS (KAKENHI 26870911), NIH grant R01-MH-081990, a Royal Society Wolfson Research Merit Award, the Swire Trust, and the NIHR Biomedical Research Centre at Moorfields Eye Hospital and the UCL Institute of Ophthalmology.

Received: August 4, 2015
Revised: September 14, 2015
Accepted: September 17, 2015
Published: October 15, 2015

REFERENCES

1. Hillis, J.M., Watt, S.J., Landy, M.S., and Banks, M.S. (2004). Slant from texture and disparity cues: optimal cue combination. J. Vis. *4*, 967–992.
2. Ernst, M.O., and Banks, M.S. (2002). Humans integrate visual and haptic information in a statistically optimal fashion. Nature *415*, 429–433.
3. Ban, H., Preston, T.J., Meeson, A., and Welchman, A.E. (2012). The integration of motion and disparity cues to depth in dorsal visual cortex. Nat. Neurosci. *15*, 636–643.
4. Dövencioğlu, D., Ban, H., Schofield, A.J., and Welchman, A.E. (2013). Perceptual integration for qualitatively different 3-D cues in the human brain. J. Cogn. Neurosci. *25*, 1527–1541.
5. Murphy, A.P., Ban, H., and Welchman, A.E. (2013). Integration of texture and disparity cues to surface slant in dorsal visual cortex. J. Neurophysiol. *110*, 190–203.
6. Gu, Y., Angelaki, D.E., and Deangelis, G.C. (2008). Neural correlates of multisensory cue integration in macaque MSTd. Nat. Neurosci. *11*, 1201–1210.
7. Nardini, M., Jones, P., Bedford, R., and Braddick, O. (2008). Development of cue integration in human navigation. Curr. Biol. *18*, 689–693.
8. Gori, M., Del Viva, M., Sandini, G., and Burr, D.C. (2008). Young children do not integrate visual and haptic form information. Curr. Biol. *18*, 694–698.
9. Nardini, M., Bedford, R., and Mareschal, D. (2010). Fusion of visual cues is not mandatory in children. Proc. Natl. Acad. Sci. USA *107*, 17041–17046.
10. Kiorpes, L. (2015). Visual development in primates: neural mechanisms and critical periods. Dev. Neurobiol. *75*, 1080–1090.
11. Norcia, A.M., and Tyler, C.W. (1985). Spatial frequency sweep VEP: visual acuity during the first year of life. Vision Res. *25*, 1399–1408.
12. Braddick, O.J., and Atkinson, J. (2009). Infants' sensitivity to motion and temporal change. Optom. Vis. Sci. *86*, 577–582.
13. Dupont, P., De Bruyn, B., Vandenberghe, R., Rosier, A.M., Michiels, J., Marchal, G., Mortelmans, L., and Orban, G.A. (1997). The kinetic occipital region in human visual cortex. Cereb. Cortex *7*, 283–292.
14. Larsson, J., and Heeger, D.J. (2006). Two retinotopic visual areas in human lateral occipital cortex. J. Neurosci. *26*, 13128–13142.
15. Popple, A.V., Smallman, H.S., and Findlay, J.M. (1998). The area of spatial integration for initial horizontal disparity vergence. Vision Res. *38*, 319–326.
16. Wallace, M.T., Perrault, T.J., Jr., Hairston, W.D., and Stein, B.E. (2004). Visual experience is necessary for the development of multisensory integration. J. Neurosci. *24*, 9580–9584.
17. Xu, J., Yu, L., Rowland, B.A., Stanford, T.R., and Stein, B.E. (2012). Incorporating cross-modal statistics in the development and maintenance of multisensory integration. J. Neurosci. *32*, 2287–2298.
18. Rogers, B., and Graham, M. (1982). Similarities between motion parallax and stereopsis in human depth perception. Vision Res. *22*, 261–270.
19. Howard, I.P. (2012). Perceiving in Depth, Volume 3: Other Mechanisms of Depth Perception (Oxford University Press).
20. Petrini, K., Remark, A., Smith, L., and Nardini, M. (2014). When vision is not an option: children's integration of auditory and haptic information is suboptimal. Dev. Sci. *17*, 376–387.
21. Yu, L., Rowland, B.A., and Stein, B.E. (2010). Initiating the development of multisensory integration by manipulating sensory experience. J. Neurosci. *30*, 4904–4913.
22. Fair, D.A., Dosenbach, N.U.F., Church, J.A., Cohen, A.L., Brahmbhatt, S., Miezin, F.M., Barch, D.M., Raichle, M.E., Petersen, S.E., and Schlaggar, B.L. (2007). Development of distinct control networks through segregation and integration. Proc. Natl. Acad. Sci. USA *104*, 13507–13512.
23. Fair, D.A., Cohen, A.L., Power, J.D., Dosenbach, N.U., Church, J.A., Miezin, F.M., Schlaggar, B.L., and Petersen, S.E. (2009). Functional brain networks develop from a "local to distributed" organization. PLoS Comput. Biol. *5*, e1000381.
24. Gogtay, N., Giedd, J.N., Lusk, L., Hayashi, K.M., Greenstein, D., Vaituzis, A.C., Nugent, T.F., 3rd, Herman, D.H., Clasen, L.S., Toga, A.W., et al. (2004). Dynamic mapping of human cortical development during childhood through early adulthood. Proc. Natl. Acad. Sci. USA *101*, 8174–8179.
25. Oruç, I., Maloney, L.T., and Landy, M.S. (2003). Weighted linear cue combination with possibly correlated error. Vision Res. *43*, 2451–2468.
26. Skoczenski, A.M., and Norcia, A.M. (1998). Neural noise limitations on infant visual sensitivity. Nature *391*, 697–700.
27. Buss, E., Hall, J.W., 3rd, and Grose, J.H. (2009). Psychometric functions for pure tone intensity discrimination: slope differences in school-aged children and adults. J. Acoust. Soc. Am. *125*, 1050–1058.
28. Beck, J.M., Ma, W.J., Pitkow, X., Latham, P.E., and Pouget, A. (2012). Not noisy, just wrong: the role of suboptimal inference in behavioral variability. Neuron *74*, 30–39.
29. Friston, K. (2010). The free-energy principle: a unified brain theory? Nat. Rev. Neurosci. *11*, 127–138.
30. Clark, A. (2013). Whatever next? Predictive brains, situated agents, and the future of cognitive science. Behav. Brain Sci. *36*, 181–204.
31. Yoon, J., Winawer, J., Witthoft, N., and Markman, E. (2007). Striking deficiency in top-down perceptual reorganization of two-tone images in preschool children. In Proceedings of the IEEE 6th International Conference on Development and Learning, 181–186.
32. Bova, S.M., Fazzi, E., Giovenzana, A., Montomoli, C., Signorini, S.G., Zoppello, M., and Lanzi, G. (2007). The development of visual object recognition in school-age children. Dev. Neuropsychol. *31*, 79–102.
33. Dekker, T., Mareschal, D., Sereno, M.I., and Johnson, M.H. (2011). Dorsal and ventral stream activation and object recognition performance in school-age children. Neuroimage *57*, 659–670.
34. Ellemberg, D., Hansen, B.C., and Johnson, A. (2012). The developing visual system is not optimally sensitive to the spatial statistics of natural images. Vision Res. *67*, 1–7.
35. Kriegeskorte, N., Goebel, R., and Bandettini, P. (2006). Information-based functional brain mapping. Proc. Natl. Acad. Sci. USA *103*, 3863–3868.
36. Chang, C.-C., and Lin, C.-J. (2011). LIBSVM: a library for support vector machines. ACM Trans. Intell. Syst. Technol. *2*, 1–27.

Current Biology

Supplemental Information

Late Development of Cue Integration Is Linked to Sensory Fusion in Cortex

Tessa M. Dekker, Hiroshi Ban, Bauke van der Velde, Martin I. Sereno,
Andrew E. Welchman, and Marko Nardini

Figure S1, relates to Figure 3

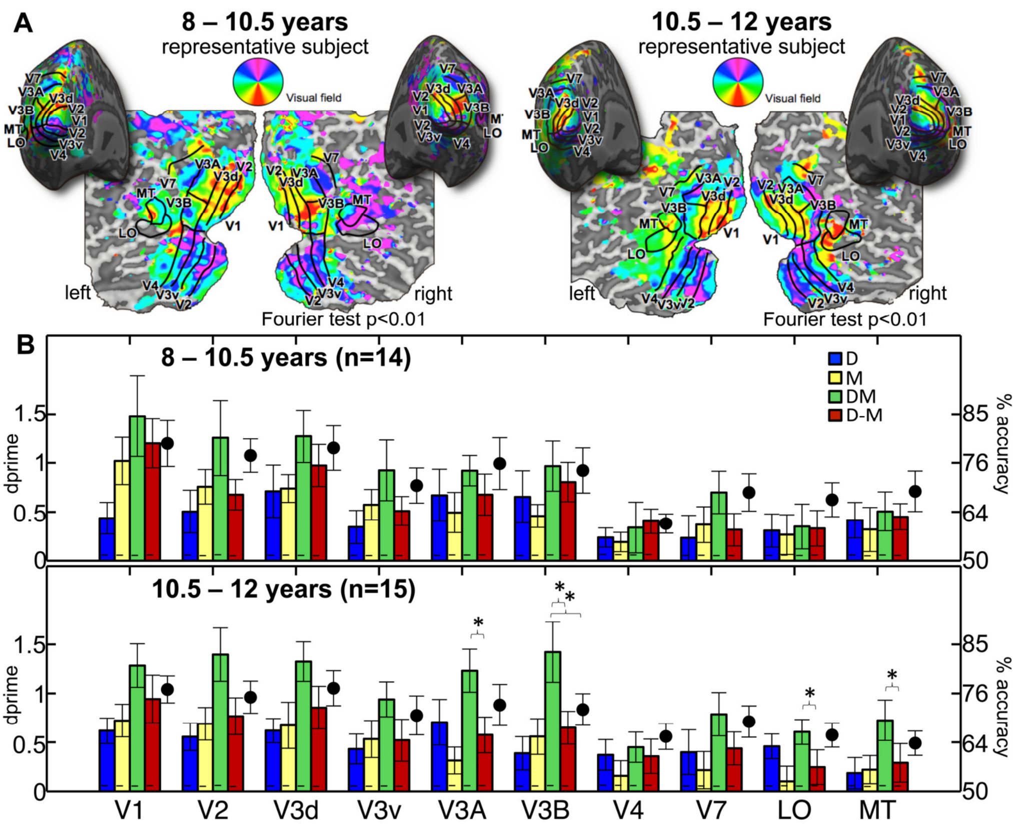

(**A**) Polar angle maps and borders of retinotopic regions of a representative younger and older subject are displayed on the same subjects' inflated and flattened cortical surfaces. (**B**) Depth decoding performance (d-prime and accuracy) for all retinotopic regions of interest is plotted per condition and age group. Error bars: 95%CI. Stars indicate p>0.05, Bonferroni-corrected, for description of analysis and p-values see Table S1.

We note that there is a (non-significant) trend for decoding for combined cues (DM) to be higher than for single cues (D or M) and conflicting cues (D-M) in visual ROIs along the cortical hierarchy – although only area V3B showed robust evidence for age-related changes in sensory fusion. This pattern differs somewhat from that seen by Ban et al. This may be (i) due to differences across experimental set-ups, or (ii) because aspects of sensory fusion are still developing between the ages of 10.5-12 years (as seen here) and adulthood (as reported previously). For example, initially, low-level areas may be recruited when children first start combining cues, and processing may gradually become more focused around area V3B. While we observe a clear shift in cortical function around the age when perceptual changes in sensory integration occur, it would be interesting to track development further, from age 10-12 years to adulthood to distinguish these possibilities.

Figure S2, relates to Figure 3

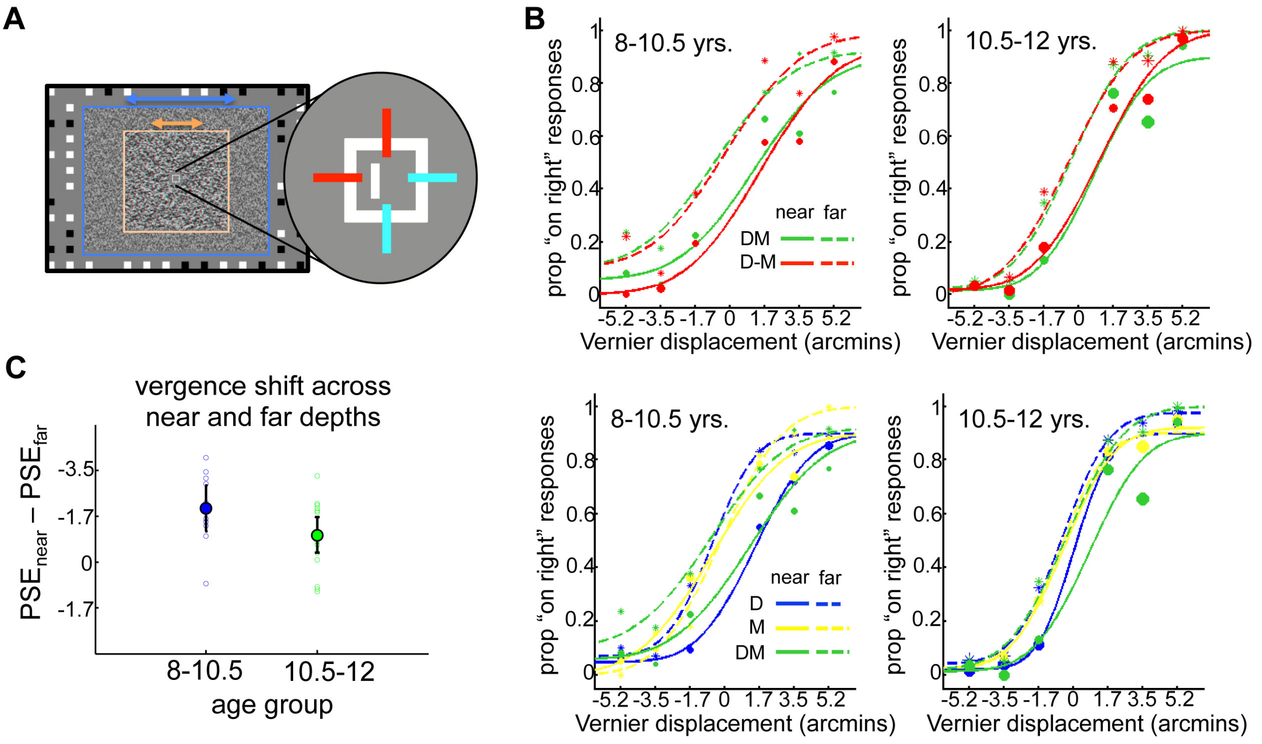

(**A**) During the Vernier task in the scanner, subjects judged whether targets (white stripe) appeared to the left or right of the top red nonius line. Targets appeared once every 4 trials (twice per block) on average, at three distances left or right of the centre. Because the red line is only presented to the left eye, a biased point of subjective equality (PSE, 50% "on the right" responses) indicates vergence in front of (positive) or behind (negative) the screen. (**B**) To compare vergence shifts across age, we fitted individual psychometric functions (Cumulative Gaussians) through responses for near and far planes collapsed across all conditions, and computed the difference in PSE for each individual (plotted, mean ± 95%CI). Four participants in the youngest group were excluded from the analysis, due to recording error (1) or because the functions fitted through their responses had a poor fit (GoF < 0.7). Subjects shifted their vergence across near and far stimuli by ~2 arcmins, a small proportion (~15%) of the total depth difference between the stimuli (12 arcmins). A trend for less vergence adjustment in the older age group did not reach statistical significance ($t(23)=1.83$, $p=0.079$). Crucially, there were no significant correlations between shifts in vergence and pattern classifier performance in any of the ROIs (largest Pearson's $r=0.30$, $p=0.15$, in area V2, condition D), suggesting that vergence shifts did not drive voxel activation patterns, and hence are an unlikely explanation of our results. **(C)**

Because there were not enough datapoints, we could not fit functions for each condition separately for each subject. To ensure that collapsing near and far trials across conditions was valid (vergence shifts were not opposite for some conditions) we obtained psychometric functions per condition by collapsing across all subjects within each group. In both groups, the PSE shifted in a consistent way across near and far planes for the D, DM and D-M conditions (**B**, top and bottom plots). Vergence did not shift across depths defined by motion (yellow lines).

Figure S3, relates to Figure 3

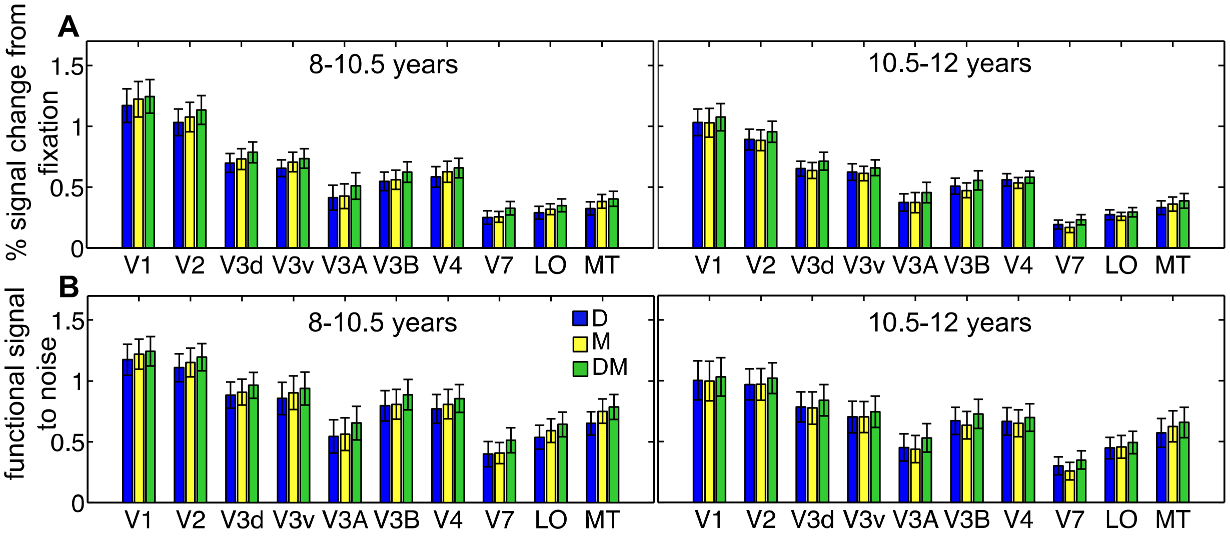

(**A**) Percent (%) fMRI signal change compared to the fixation baseline in the D, M and DM conditions, is visualized for each region of interest and age group (error bars: 95% CI). This was calculated from the mean response of the 400 voxels used for classification in each area. (**B**) To obtain a functional index of signal to noise, signal change was expressed in relation to the overall standard deviation of the signal $X_{stimulus}$- $X_{fixation}$/ $SD_{stimulus\ +\ fixation}$ (error bars: 95% CI). Both signal indices show a similar pattern: ROI (10) x Condition (3) x Age (2) ANOVAs revealed that both measures differed across stimulus conditions D, M and DM, (% signal change: $F(2,26)=29.5$, $p<0.01$; signal to noise: $F(2,26)=30.7$, $p<0.01$), with slightly higher signal for DM stimuli. Crucially, there were no significant main effects of Age (% signal change: $F(1,27)=2.1$, $p=0.16$; signal to noise: $F(1,27)=1.7$, $p=0.21$), or interactions of Age with Condition (% signal change: $F(2,26)=2.1$, $p=0.14$; signal to noise: $F(2,26)=1.9$, $p=0.17$); or with Age, Condition and ROI (% signal change: $F(5.9,156)=0.78$, $p=0.58$; signal to noise: $F(5.9,161.5)=0.39$, $p=0.88$). Furthermore, neither % signal change nor signal to noise were correlated with classifier performance in V3B, the area in which we found robust age differences in indices of sensory integration (largest Pearson's $r=0.13$, $p=0.50$). In sum, it is unlikely that age differences in our pattern classifier analysis were driven by differences in overall signal amplitude or functional signal to noise.

Table S1, relates to Figure 2.

Function Name	Description	Mean BIC
**Linear**	f(x) = p1*x + p2	-90.193
**Exponential**	**f(x) = p1*exp(p2*x)**	**-92.731**
**Quadratic**	f(x) = p1*x^2 + p2*x + p3	-92.179
**Two lines with inflection** Least square splines, 3 knots	Slope1=0, slope 2=free	-92.090
**Two lines with inflection** Least square splines, 3 knots	Slope1=free, slope 2=free	-88.980

To assess which function best captures the change in the integration indices in Figure 2A, we compared 5 plausible models: (1) a straight line, (2) an exponential increase, and (3) a quadratic change. We also fit the data with two straight lines connected by an inflection point using least square splines [S1], with (4) the slope of the first line set to zero (intercept, inflection-point and second slope free parameters), or (5) a freely varying first slope. For model comparison we used the Bayes Information Criterion, where the lowest value indicates the best-fitting model after model complexity is accounted for (i.e., models with more parameters are less parsimonious, so penalised). The best-fitting model was an exponential function. For DM vs. D-M: p1=$7.25*10^{-05}$ (95% CI: $-0.3152*10^{-4}$, $4.603*10^{-4}$) p2=0.6201 (95% CI: 0.1776, 1.062), R^2=0.10. For DM vs. $\sqrt{}D^2+M^2$: p1=$2.152*10^{-6}$ (95% CI: -2.43e-05, 2.861e-05), p2=0.8641, (95% CI: -0.1314, 1.86), R^2=0.15. The resulting curves (**black, Fig. 2A**) are in line with previous reports of no integration followed by integration in childhood [S2–S5], and with a gradual developmental process (not all children start integrating at exactly the same age). It is important to note, however, that model 3 (a quadratic function with a slight initial decrease followed by a steeper increase) and model 4 (a flat line connecting to an increasing slope) also had good fits to the data and led to similar conclusions that robust integration emerges around age 10.5 years.

Table S2, relates to Figure 3

	**DM *vs.* D-M**			**DM *vs.* $\surd M^2+D^2$**		
	p-values			*p-values*		
	<10.5 yrs	>10.5 yrs	age diff.	<10.5 yrs	>10.5 yrs	age diff.
V1	0.28	0.21	0.87	0.32	0.10	0.90
V2	0.057	0.022*	0.89	0.49	0.069	0.46
V3v	0.22	0.043*	0.59	0.58	0.094	0.56
V3d	0.12	0.0084*	0.99	0.46	0.18	0.97
V3A	0.086	0.0045*	0.09	0.66	0.035*	0.067
V3B	0.43	0.0022*	0.04*	0.80	0.0048*	0.036*
V4	0.76	0.49	0.52	0.85	0.37	0.78
V7	0.071	0.12	0.91	0.10	0.64	0.77
LO	0.94	0.0047*	0.20	0.10	0.77	0.11
MT	0.79	0.0040*	0.11	0.27	0.22	0.095

We tested which areas met the criteria for cue integration by comparing DM vs. D-M and DM vs. $\surd(D^2+M^2)$, using paired t-tests. We also tested if the two integration indices DM - D-M and DM - $\surd(D^2+M^2)$ differed significantly across age, using independent t-tests comparing index across age (results in table, * = $p<0.05$, underlined values survived Bonferroni correction). As explained in the main report, we specifically expected age-related changes in sensory fusion in area V3B (i.e. we had planned comparisons for this area based on three previously published studies). In younger children (<10.5 years), no area met both criteria for cue integration. In older children, area V3B met both criteria, and the age difference in both integration indices was significant (based on planned comparisons). We found a similar, but only marginally significant effect in area V3A. This is not surprising because V3A may have similar functional properties as V3B (since they are directly adjacent), and because slight inaccuracies in border identification may have miss-assigned parts of V3B to V3A.

Supplemental Experimental Procedures

Selection procedures for MRI

We first tested a large number of subjects between ages 6-12 years behaviourally in our lab and a local school (76; 50 of whom met inclusion criteria and are included in Fig 2). We observed an increase in integration between ages 8-12 years in this group. We then used identical procedures to test 67 more children in this smaller age range (53 of whom met inclusion criteria and are included in Fig 2), with the intention to select subjects for MRI. We invited 41 children back for fMRI (29 of whom met inclusion criteria - magenta data points in Fig 2, and data in Fig 3). They were selected based on several considerations: (1) parents *and* children were willing to take part after the experimenter explained what fMRI involved, (2) families were available during scanning slots within ~6 weeks of behavioural testing, (3) children met MRI safety criteria, (4) children appeared to not be very fidgety. We also attempted to (5) obtain a representative sample of each age group <10.5 & above >10.5 in terms of overall integration performance, and we aimed to (6) obtain age groups of equal size with good fMRI data quality (~15 subjects >10.5 years and <10.5 years).

To ensure that age differences were not driven by age-related confounds in attention, compliance, etc., we had to prioritise data quality over inclusion. The pattern of results stayed similar with more lenient exclusion criteria (see "Participants" section in methods). We judge it unlikely that a basic perceptual function (i.e., sensory fusion) is sensitive to any population sampling biases this may have caused.

Rewards for behavioral performance

To keep child participants motivated, focussed and entertained throughout the experimental tasks, they were shown their score at the end of each experimental block (or run inside the scanner), creating a game-like element. Scores were converted into coins that could be used to "buy" small prizes at the end of the session. The number of coins won was indicated by the number of smiley faces next to the interval in which the score fell along a score-bar from 0-100. Although participants were not informed of this, we computed scores based on the easiest trials only, to reward attention rather than individual differences in perceptual ability.

Procedures fMRI session 1

Before collecting pattern classification fMRI data, we collected a high-resolution structural scan and retinotopic data from each subject in a separate fMRI session. BrainVoyager QX (BrainInnovation B.V.) was used to transform each subject's T1-weighted 3D MPRAGE (1 mm^3 voxel size, Bandwidth=190 Hz/pix, 176 partitions, TR=8.4, TE=3.57, effective TI=1000 ms, flip angle=7 degrees) into Talairach space, inflate the cortex and create flattened surfaces of both hemispheres. We obtained retinotopic maps by measuring BOLD responses whilst subjects were presented with a rotating wedge stimulus overlaid with moving objects (polar angle mapper, cycles/run=6, eccentricity=~16°, 2 runs with clockwise, 2 counter-clockwise rotation). To stimulate fixation, subjects performed a central task: They detected brightness changes of the fixation dot during runs 1 and 2, and they detected letters briefly flashed on top of the dot during runs 3 and 4. We used a standard EPI sequence (TR=2.5s, volumes=132, slices=30 voxel size=3.2 cm^3, axial plane, interleaved, bandwidth=1930 Hz/pix, TE= 39 ms, flip=90).

Pre-procession steps session 1 and 2

Preprocessing steps for all functional data collected during MRI sessions 1 (retinotopic mapping) and 2 (depth cue integration), consisted of: initial volume removal (4 TRs), motion correction, slice time correction, linear trend removal and high-pass filtering (three cycles per run cut-off). We aligned functional runs to each subject's anatomical scan and both were transformed into Talairach space.

ROI selection

We used Fourier analyses and Fourier F-tests to obtain polar angle phase maps, which were then projected onto the subject's inflated cortical surface. Borders of visual areas of interest were identified for each individual following criteria derived from Ban et al. (2012). Previously, V3B/KO was mapped using a dedicated localiser. Due to limitations on scanning with young children, V3B was defined based on retinotopy alone. We identified the first appearance of upper field in superior visual cortex bordering V3, and estimated where the eccentricity minimum was between V3A and V3B [S6]. We then extended V3B down to the centre of gaze. This overlapped our V3B with an area that has been described as LO1 (e.g., [S7], and was activated by our V3B/KO localiser. The location of LO was informed by an object vs. scrambled object localiser Ban et al., 2012 did with adults, and follows [S8].

Details of Searchlight analyses

The searchlight analysis procedures are similar to those reported in [S9], to allow for comparison to adult data. Specifically, we performed the searchlight analysis in volume space for each individual by selecting spherical ROI with 8mm radii, moving voxel-wise through the entire scanned volume of cortex. To account for variability in spatial organisation across subjects, each accuracy map was then smoothed with a 3mm FWHM kernel to reduce inter-subject variability. We computed group-level t-value maps by testing where $DM>D-M$ and $DM>\sqrt{(D^2+M^2)}$ (conjunctive $p<0.05$), and projected the result onto a representative cortical surface from each group for visualisation (shown in Figure 3). The results from the searchlight analysis are highly consistent with the results from the ROI analyses, which effectively factors out individual differences in retinotopic organisation. The results of older children (>10.5 years) are consistent with previous results from adults [S9]. When we aligned subjects into a common space based on gyral and sulcal patterns along the cortex [S10], respecting V3B ROI locations (following function-informed cortex-based alignment procedures described in Frost & Goebel, 2013), the searchlight results remained similar, with a potential slight shift towards V3A in the right hemisphere, consistent with marginally significant fusion effects in the V3A ROIs (see p. 5, and caption Figure 1S).

Supplemental References

1. D'Errico (2009). Matlab slmengine toolbox: http://www.mathworks.com/matlabcentral/fileexchange/24443-slm-shape-language-modeling.

2. Gori, M., Del Viva, M., Sandini, G., and Burr, D. C. (2008). Young children do not integrate visual and haptic form information. Curr. Biol. *18*, 694–698.

3. Nardini, M., Jones, P., Bedford, R., and Braddick, O. (2008). Development of cue integration in human navigation. Curr. Biol. *18*, 689–693.

4. Nardini, M., Bedford, R., and Mareschal, D. (2010). Fusion of visual cues is not mandatory in children. Proc. Natl. Acad. Sci. *107*, 17041.

5. Petrini, K., Remark, A., Smith, L., and Nardini, M. (2014). When vision is not an option: children's integration of auditory and haptic information is suboptimal. Dev. Sci. *17*, 376–387.

6. Press, W. A., Brewer, A. A., Dougherty, R. F., Wade, A. R., and Wandell, B. A. (2001). Visual areas and spatial summation in human visual cortex. Vision Res. *41*, 1321–1332.

7. Larsson, J., and Heeger, D. J. (2006). Two retinotopic visual areas in human lateral occipital cortex. J. Neurosci. Off. J. Soc. Neurosci. *26*, 13128–13142.

8. Malach, R., Reppas, J. B., Benson, R. R., Kwong, K. K., Jiang, H., Kennedy, W. A., Ledden, P. J., Brady, T. J., Rosen, B. R., and Tootell, R. B. (1995). Object-related activity revealed by functional magnetic resonance imaging in human occipital cortex. Proc. Natl. Acad. Sci. *92*, 8135–8139.

9. Ban, H., Preston, T. J., Meeson, A., and Welchman, A. E. (2012). The integration of motion and disparity cues to depth in dorsal visual cortex. Nat. Neurosci. *15*, 636–643.

10. Fischl, B., Sereno, M. I., Tootell, R. B., and Dale, A. M. (1999). High-resolution intersubject averaging and a coordinate system for the cortical surface. Hum. Brain Mapp. *8*, 272–284.
